# The effect of radio-frequency ablation in treating pulmonary ground glass nodule patients and its influence on pulmonary function

**DOI:** 10.4314/ahs.v24i4.32

**Published:** 2024-12

**Authors:** Chengwei Zhou, Zixuan Chen, Yuan Zhang, Xiaodong Zhao

**Affiliations:** 1 Department of Thoracic Surgery, The Affiliated Hospital of Medical School of Ningbo University, Ningbo, China; 2 Department of PCCM, Zhenhai Hospital of TCM Ningbo, Ningbo, China

**Keywords:** Radio-frequency ablation, pulmonary ground glass nodule, pulmonary function, inflammatory cytokines

## Abstract

**Background:**

With widespread application of low-dose thin-section chest CT screening, the detection rate of patients with subsolid nodules (SNs) as CT findings has increased remarkably.

**Objective:**

To clarify effect of radio-frequency ablation (RFA) in treating pulmonary ground glass nodule (GGN) patients and its impact on pulmonary function.

**Methods:**

A total of 100 patients diagnosed with pulmonary GGN in our hospital from January 2019 to December 2021 by pulmonary thin-section CT + enhanced imaging examination and all underwent simultaneous needle biopsy were enrolled and randomly divided into control group and observation group, with 50 cases each. The control group received treatment with traditional surgery. The observation group received treatment with RFA. The surgical indicators, surgical treatment efficacy, levels of inflammatory cytokines, complications and pulmonary function indicators were assessed between two groups.

**Results:**

The operation time, hospital stay and intraoperative blood loss in observation group were markedly shorter/less than controls (P < 0.05). No residual recurrence was observed in total 84 nodules of 50 cases in observation group. The size of nodules was observed by CT 1, 3 and 6 months after operation. With prolongation of postoperative time, nodules gradually shrank, after 6 months, fibrous cord scar residue gradually formed, with statistical significance relative to those before operation (P < 0.05). The postoperative IL-6, CRP and TNF-α in both groups presented elevation, whereas postoperative changes in IL-6, CRP and TNF-α in observation group presented depletion relative to control group, with statistical significance (P < 0.05). The complication rate in observation group presented depletion relative to control group, with statistical significance (P < 0.05). The FVC, FEV1, FEV1%, MVV and PEF in both groups presented depletion 1 month after operation and presented statistical significance relative to those before operation except for FEV1% (P < 0.05), and changes in observation group presented depletion relative to control group. Six months after operation, FVC, FEV1, FEV1%, MVV and PEF in both groups had recovered to preoperative levels and presented no difference relative to preoperative level (P > 0.05), and presented no difference between two groups (P > 0.05).

**Conclusion:**

RFA for the therapy of pulmonary GGN is safe and effective, without surgical scar, and is less traumatic to the body, which has a good application prospect.

## Introduction

With widespread application of low-dose thin-section chest CT screening, the detection rate of patients with subsolid nodules (SNs) as CT findings has increased remarkably. Pulmonary SNs are focal, nodular, patchy, thin ground-glass opacities with elevated density in the lung as main imaging features, and original structures such as blood vessels and airways are still visible. According to whether it contains a solid component, it can be divided into ground-glass lesions and mixed ground-glass lesions^1^. Pathologically, it can manifest as focal interstitial fibrosis, infection, hemorrhage, edema, atypical adenomatous hyperplasia (AAH), adenocarcinoma in situ (AIS), minimally invasive adenocarcinoma (MIA), invasive adenocarcinoma (IA), etc.^2^, ^3^. Such lesions are small in size and difficult to diagnose on imaging, and surgical resection is the major treatment. Radical surgical procedures include traditional surgical resection and thoracoscopic resection. Traditional surgery has large damage and many complications, and exerts a great impact on pulmonary function. Thoracoscopic surgery preserves integrity of thorax and causes little damage to patients' pulmonary function. Nevertheless, for small lesions, it is difficult to locate and difficult to operate. SNs are often multiple and often occur in bilateral lungs. Due to limitation of pulmonary functional reserve, multiple thoracoscopic localized resections are difficult to implement. Thus, green and minimally invasive ablation treatment technology has been developed rapidly, especially temperature ablation treatment technology represented by radio-frequency ablation (RFA) is increasingly applied to clinical practice, providing a new option for therapy of pulmonary SNs^4^, ^5^. RFA is less invasive than surgery and can be applied repeatedly, suitable for therapy of multiple lung lesions and metastases^6^. Nevertheless, there are few domestic reports to clarify size of trauma to the body. The research attempted to clarify effect of RFA in treating pulmonary ground glass nodule (GGN) patients and its influence on pulmonary function.

## Materials and methods

### General data

A total of 100 patients diagnosed with pulmonary GGN in our hospital from January 2019 to December 2021 by pulmonary thin-section CT + enhanced imaging examination and all underwent simultaneous needle biopsy were enrolled and randomly divided into control group and observation group, with 50 cases each. Among them, there were 12 cases of multiple pulmonary nodules and 38 cases of solitary pulmonary nodules in the control group, including 23 males and 27 females, aged 35-81 years old, mean: (60 ± 13.2) years old; 8 cases with chronic obstructive pulmonary disease (COPD) and 25 cases with smoking history; a total of 82 pulmonary GGNs were ablated, with a maximum diameter of 17.8 mm, mean: (9.1 ± 3.6) mm. There were 13 cases of multiple pulmonary nodules and 37 cases of solitary pulmonary nodules in observation group, including 25 males and 25 females, aged 36-79 years old, mean: (59.3 ± 9.8) years old; 6 cases with chronic obstructive pulmonary disease (COPD) and 26 cases with smoking history; a total of 84 pulmonary GGNs were ablated, with a maximum diameter of 18.2 mm, mean: (9.3 ± 3.1) mm. General data presented no difference between two groups (P > 0.05), and they were comparable.

### Inclusion and exclusion criteria

**Inclusion criteria:** 1) With typical malignant imaging signs such as lobulation sign, burr sign, pleural depression sign, vascular cluster sign, etc.; 2) after long-term standardized follow-up, malignant transformation manifestations such as enlargement of 2 mm, increase of solid components, etc.; 3) synchronous multiple ground-glass nodules (SMGNs).

**Exclusion criteria:** 1) Suspected metastasis of lung cancer (LC), lymphatic metastasis or distant metastasis indicated by lung CT enhancement or PET/CT; 2) emphysema and bullae were severe, it was estimated that pneumothorax was unavoidable, and puncture operation could not be completed; 3) very poor cardiopulmonary function, unable to take care of themselves; 4) uncorrectable coagulation dysfunction and severe bleeding tendency; 5) patients with disturbance of consciousness or unable to cooperate with treatment, complicated with mental illness, etc.

## Methods

The control group received treatment with traditional surgery. The observation group received treatment with RFA. The surgical procedure was as follows:

1) All patients underwent preoperative chest thin-slice CT scan + enhanced scan and pulmonary function examination, and no abnormal lymph nodes were found on CT enhancement in all cases. Depending on location of nodule shown on preoperative chest CT, patients were placed in the supine, prone, or lateral position to obtain best puncture approach, and a 2.5 mm slice thickness was routinely used for CT scan.
2) For single pulmonary nodule lesions, path of biopsy needle and ablation needle was supposed to be determined after CT scanning; labelling, disinfection, drape, local anesthesia with 2% lidocaine; needle was inserted step by step, positioning needle and ablation needle were respectively advanced to reach predetermined position; ablation needle was connected to 250 ml of normal saline and host emission source system through peristaltic pump and cooling circulating water pipe; after replacement with a disposable semi-automatic biopsy gun by positioning needle, 2-3 pathological tissues were obtained; host emission source system was started, ablation power was generally 40-70 W, ablation time was 4-8 min, and ablation diameter range was about 2.0-4.0 cm; during ablation process, CT scan was supposed to be performed immediately to confirm ablation focus range, and operation would be terminated if ablation range was sufficient.
3) For SMGN patients, for multiple pulmonary nodules in single lung lobe, through interpretation and evaluation of preoperative CT, only one of the larger or relatively safe GGNs was chosen for needle biopsy; for remaining lesions, corresponding marks and points could be determined on body surface according to their positions, and puncture and ablation could be performed in the same way as above.
4) Ablation needle was pulled out after ablation, and chest CT was immediately reviewed to confirm ablation scope of pulmonary nodule and whether it was off-target. Ablation was supplemented if necessary, and whether there were complications was observed.
5) The chest thin-section CT and pulmonary function were re-examined 3 days and half a year after operation.

### Blood specimen collection and processing

The 5 ml of cubital venous blood was drawn with a sterile dry test tube on an empty stomach at 8-10 am in the morning 1 day before operation and 12 h after operation, respectively. After standing for 10 min, blood was centrifuged at 2000 r/min for 10 min to separate serum, and serum was stored in a -80°C refrigerator for testing. IL-6, TNF-α and CRP contents were detected by enzyme-linked immunosorbent assay (ELISA) kits (Ebioscience, USA), and experimental steps were referred to instructions of kits.

### Observation indicators

1). Surgical indicators. Surgical indicators included operation time (from skin incision to withdrawal), hospital stay and intraoperative blood loss (aspirator bottle suction volume minus irrigation volume).
2). Evaluation of surgery efficacy. The curative efficacy was judged through CT scan during operation: If a ground-glass opacity with a width of at least 0.5-1.0 cm appeared on periphery of original lung GGN, the lesion was considered to be completely ablated. Postoperative follow-up was for 1-6 months, mean: (4.9 ± 1.2) months. The imaging dynamic changes of lesions after pulmonary GGN ablation were assessed by regular thin-section CT scans, and local recurrence of ablation lesions was observed. Nodule volume (V = πabc / 6, where V was nodule volume, a was the largest diameter, and b and c were the other two mutually perpendicular diameters). Nodule shrinkage rate: [(volume before treatment - volume at follow-up) / volume before treatment] × 100%.
3). Postoperative recovery inflammatory indicators. IL-6, TNF-α and CRP contents were detected 1 day before operation and 12 h after operation.
4). The incidence of complications. Proportion of number of patients with various complications to total number of patients (complications) during postoperative hospital recovery period.
5). Pulmonary function assessment. The patients in both groups underwent regular re-examination of pulmonary function 1 month and 6 months after surgery, including forced vital capacity (FVC), forced expiratory volume in one second (FEV1), FEV1 as a percentage of predicted (FEV1%), maximal voluntary ventilation (MVV) and peak expiratory flow (PEF).

### Statistical analysis

Normally distributed measurement data were expressed as mean ± standard deviation (x ± s). SPSS 20.0 software was used for statistical analysis. Independent samples t test was used for comparison between groups, and X2 test was used for count data and rate comparison. P < 0.05 was considered statistically significant.

## Results

### Comparison of surgical indicators between two groups

**Figure 1:**
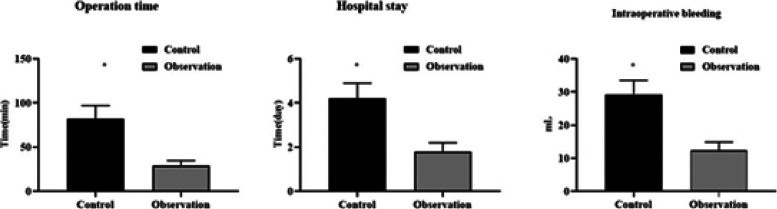
Surgical indicators in two groups

The operation time, hospital stay, and intraoperative blood loss in observation group were markedly shorter/less than controls (P < 0.05, Figure 1).

### Comparison of treatment efficacy between two groups

**Figure 2:**
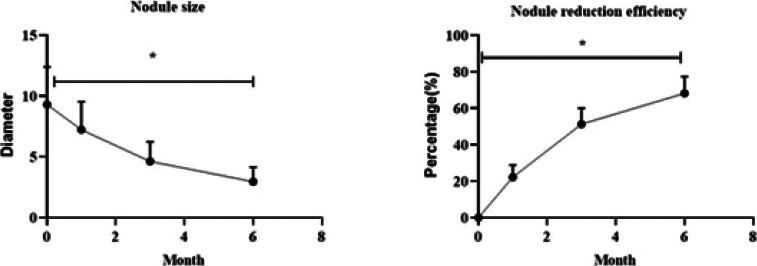
Evaluation of treatment efficacy of patients

No residual recurrence was observed in total 84 nodules of 50 cases in observation group. The size of nodules was observed by CT 1, 3 and 6 months after operation. With prolongation of postoperative time, nodules gradually shrank, and after 6 months, fibrous cord scar residue gradually formed, with statistical significance relative to those before operation (P < 0.05, Figure 2).

### Comparison of postoperative recovery inflammatory cytokine levels between two groups

**Figure 3:**
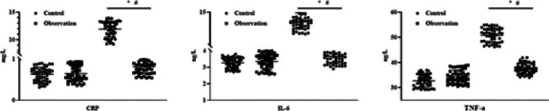
Postoperative recovery inflammatory cytokine levels in two groups

The postoperative IL-6, CRP and TNF-α in both groups presented elevation, whereas postoperative changes in IL-6, CRP and TNF-α in observation group presented depletion relative to control group, with statistical significance (P < 0.05, Figure 3).

### Comparison of complication rates between two groups

The complication rate in observation group presented depletion relative to control group, with statistical significance (P < 0.05). Among complications in two groups, the incidence of pleural effusion, hemoptysis, local infection and intrapulmonary hemorrhage was exhibited in table 1.

**Table 1:** Complications in two groups

Groups	Complication				Complication rate
	Pleural effusion	Hemoptysis	Local infection	Intrapulmonary hemorrhage	
Control group	2 (4.00)	2 (4.00)	4 (8.00)	1 (2.00)	18.00
Observation group	1 (2.00)	1 (2.00)	1 (2.00)	0 (0.00)	6.00
t	5.384				
P	< 0.001				

### Comparison of pulmonary function between two groups

**Figure 4:**
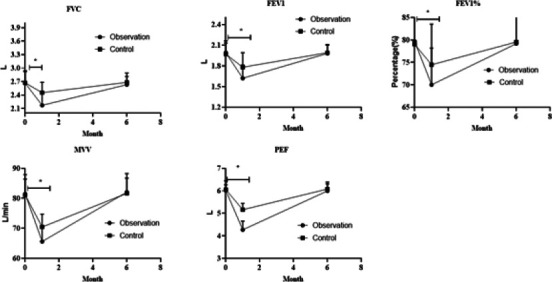
Pulmonary function of in two groups

The FVC, FEV1, FEV1%, MVV and PEF in both groups presented depletion 1 month after operation and presented statistical significance relative to those before operation except for FEV1% (P < 0.05), and changes in observation group presented depletion relative to control group; 6 months after operation, FVC, FEV1, FEV1%, MVV and PEF in both groups had recovered to preoperative levels and presented no difference relative to preoperative level (P > 0.05), and presented no difference between two groups (P > 0.05, Figure 4).

## Discussion

In recent years, incidence of LC in China has been on the rise year by year, and now it ranks first among all malignancies, among which lung adenocarcinoma (LUAD) accounts for the highest proportion. At present, 5-year survival rate of LC patients in the world is only 19%, and the reason is that most of LC patients are in the middle and advanced stages when diagnosed with LC^7^. Pulmonary GGN is currently recognized as an early sign of LUAD, and according to pathology of LUAD, it can include invasive adenocarcinoma (IA), minimally invasive adenocarcinoma (MIA) and preinvasive lesions^8^, ^9^; preinvasive lesions can be further divided into adenocarcinoma in situ (AIS) and atypical adenomatous hyperplasia (AAH)^8^. Thus, if LC can be found in preinvasive lesion or MIA stage and receive active treatment, due to the current international consensus that local excision can achieve curing^10^, in theory a radical cure can be achieved. Therefore, early diagnosis and timely therapy of suspected malignant pulmonary GGN can significantly improve prognosis of LUAD patients and prolong survival time of patients. Nevertheless, there are still some patients with pulmonary GGN who are unable or unwilling to undergo thoracoscopic surgery for various reasons, and this group of people urgently needs a relatively safe and effective alternative treatment. Herein, RFA was used to treat pulmonary GGN, and all cases achieved good results. Within a limited follow-up period, ablation foci gradually shrank and became stable, providing a new idea for clinicians to treat this kind of population.

Currently, RFA exerts an increasingly vital role in treating LC, and its application range includes non-small cell lung cancer and metastases^11^, ^12^. RFA is less invasive than surgery and can be applied repeatedly, suitable for therapy of multiple lung lesions and metastases^13^, ^14^. Pulmonary lesions dominated by SNs generally contain more air than solid lesions and thus have lower electrical conductivity than solid tumors. Nevertheless, multiple studies have revealed that its technical success rate is not lower than that of ablation of solid tumors^15^. The working principle of RFA is mainly to eliminate nodular tissue using heat energy. Radio-frequency waves radiate nodular tissue, oscillate ions in cells and generate heat through impact of its polar macromolecules. After the temperature rises to an effective stage, it can kill the diseased cells for a considerable time^16^. For instance, Kodama et al. performed RFA on 33 patients with a total of 42 lesions mainly composed of SNs. By comparing patients' survival time and complications, they discovered that RFA is a feasible, safe and effective treatment for LUAD with predominantly SN components, and no disease-related death occurred in all patients within 5 years^17^. Iguchi et al. performed RFA treatment on 16 LC patients with SN and found that no major complications occurred in all patients, and no patients had disease-related death within 5 years after surgery 18, strongly confirming efficacy and safety of RFA in treating LC with SN. As RFA uses heat energy to cause coagulation and necrosis of tissue and self-absorption, it will cause certain trauma to the body just like traditional surgery. After operation, various complex stress responses will occur in the body, and various cytokines such as IL-6, CRP, TNF-α, etc., will change. Herein, we compared effects of two operations on body trauma. The observation group presented less intraoperative blood loss, shorter operation time and shorter postoperative hospital stay than controls. Postoperative complication rates presented statistical significance between two groups.

In the acute phase reaction stage after trauma, IL-6 produced by monocytes, fibroblasts, lymphocytes and endothelial cells can induce hepatocytes to synthesize CRP. At present, it is believed that complex changes of cytokines in vivo after operation also have relation to size of surgical trauma^19^. IL-6 gets main involvement in early postoperative inflammatory response, and shows a marked elevation after surgical trauma. The increase has relation to degree of injury of operation itself, which can be used to determine early trauma stress intensity^20^. CRP is synthesized by hepatocytes under induction of IL-6. Postoperative serum CRP response level has positive association with size of surgical injury, which is a vital sensitive indicator for reflecting degree of surgical injury^21^. TNF-α can be used as a marker of cytokine secretion and is an early sensitive marker of tissue damage^22^. Herein, changes of postoperative trauma stress response indicators, CRP, IL-6, and TNF-α, in observation group presented depletion relative to control group, with statistical significance (P < 0.05), and intraoperative blood loss, operation time and postoperative hospital stay presented statistical significance between two groups (P < 0.05), suggesting that though ablation by heat conduction has a certain degree of thermal damage to the body, the damage degree is remarkably smaller than that of traditional surgery. After regular follow-up in observation group, nodule tissue gradually got degenerated and necrotic, and nodules were absorbed by phagocytic function of body. After 1, 3, and 6 months of follow-up, median nodule shrinkage rates were 22.2%, 51.3% and 68.2%, respectively, achieving very satisfactory results. Although not all patients' nodules can be completely regressed, but nodules have been inactivated and its possibility of canceration is extremely small. Thus, RFA can be more precise and minimally invasive to achieve purpose of therapy.

Herein, dynamic follow-up and comparison of pulmonary function before and after pulmonary GGN ablation allowed us to have a further understanding of influence on pulmonary function after microwave ablation of pulmonary GGN. All cases were re-examined 3 days after operation. The influence on individual patients' pulmonary function was small, but on most of patients was remarkable. Statistical analysis demonstrated that pulmonary function indicators in short term are worse than those before operation. FVC, FEV1, MVV and PEF are obvious, which may have relation to factors such as lung tissue damage, pain at the puncture site, a small amount of pneumothorax, intrapulmonary hemorrhage, pleural effusion, etc., after lung ablation. Herein, there were 4 patients with COPD, the lowest FEV1% was 43.19%, and there was no postoperative asthma exacerbation, whereas FEV1% did not change much before and after ablation, also suggesting that this procedure generally does not cause airway obstruction and has less impact on patients with obstructive airway disease. The re-examination of pulmonary function after half a year demonstrated that all patients returned to preoperative state, which presents rough consistency with period of pulmonary function recovery after lung surgery. Moreover, according to dynamic follow-up of thin-layer CT scans of lungs, lesions gradually shrank with time after lung ablation. After half a year, lesions had basically evolved into fibrous cord-like scars, and they did not evolve further since then, which confirms the recovery process of lung function from the side. After half a year, pulmonary function can be completely recovered, undoubtedly providing a solid pulmonary function guarantee for the fractionated treatment of SMGNs.

In conclusion, RFA for the therapy of pulmonary GGN is safe and effective, without surgical scar, and is less traumatic to the body, which has a good application prospect.
